# Supplementary material for: Global resource potential of seasonal pumped hydropower storage for energy and water storage
Source: Nat Commun. 2020 Feb 19;11:947. doi: 10.1038/s41467-020-14555-y (PMC7031375; doi:10.1038/s41467-020-14555-y)
Supplement: Supplementary file 2 — Supplementary Information [file 41467_2020_14555_MOESM2_ESM.docx]

**Global resource potential of seasonal pumped hydropower storage**

**for energy and water storage**

Hunt et al.

**Supplementary Information**

**Supplementary Table 1**

**Conditions where SPHS could be used for energy and water storage.**

| **Challenges** | **Benefits of deploying seasonal pumped-storage** |
| --- | --- |
| **Energy storage benefits** | |
| **Highly seasonal hydropower generation**^1–3^ | - Increase water and energy storage in water basins to regulate the river flow and increase hydropower generation. - Store excess water during periods of high hydropower generation and reduce spillage. |
| **Goal for CO_2_ emissions reduction**^4–6^ | - Hydropower, solar and wind generation usually do not have the same seasonal generation profile as the demand for electricity. Natural Gas is an option for flexible electricity generation, however, it is a fossil fuel based source of energy and emits CO_2_. A seasonal storage option should be considered by countries that intend to considerably reduce CO_2_ emissions. |
| **Increase in solar power generation in countries at high latitudes** | - Countries in high latitudes have a strong seasonal solar power generation profile. Seasonal storage allows energy to be stored in the summer and generate electricity during the winter, when there is lower solar generation. |
| **Seasonal demand variations** | - Countries in mid and high latitudes tend to have a seasonal electricity demand profile. For example, they can consume more electricity in the summer for cooling or during the winter for heating purposes. Typically, the peak national grid demand can be two to three times as high as the minimum demand^7^. |
| **Electrification of the heating sector** | - With the electrification of the heating sector in countries at high latitude, the demand of electricity during the winter will increase even further. |
| **Low energy security**^8^ | - Reduction in fluctuation of electricity prices with fossil fuel prices and supply. - Reduction in fluctuation of electricity prices with renewable energy availability, especially hydropower. - Reduction in fluctuation of electricity prices with the demand for electricity. |
| **Low power plant capacity factor** | - Large part of the generation capacity of a country is on stand-by for energy security reasons. The number of stand-by plants would reduce it seasonal pumped-storage is implemented. |
| **Island electricity generation**^9,10^ | - Costs of oil and diesel based electricity generation for island electricity generation might be higher than the combination of renewable sources and energy storage. |
| **Water storage benefits** | |
| **Inappropriate topography** | - SPHS plants can store water on higher ground away from the river, in cases where along the river is infeasible |
| **High evaporation rates** | - Water storage in reservoirs with high level variation considerably reduces evaporation rates due to higher volume to area ratio. |
| **High storage reservoir sedimentation** | - SPHS projects have much smaller sedimentation rates than conventional dams due to the small catchment area. |
| **Lower environmental and social impacts**^11^ | - Damming a major river for storage would result in higher environmental and social impact than damming a small tributary river. SPHS allows water storage without fragmenting the ecosystem of a main river, assuming that a cascade of hydropower plant is not installed in the river. The implicit comparison to conventional reservoir dams tends to minimize seasonal pumped-storage environmental impacts or disadvantages. |
| **Better water quality control** | - Storing water in a SPHS reservoir parallel to the river allows for a better control of the water quality in the reservoir, as it would not be directly affected by the fluctuations in water quality in the main river. Usually the water quality of a river deteriorates when the river flow is low and there is not enough water to dilute the pollutants in the water. In these low water availability periods, the SPHS plant will not pump water from the river. Water will be pumped into the river when the river flow rate is higher and the pollutants in the water are more dissolved. This will contribute to maintaining a better water quality in the reservoir. If the SPHS is still required to provide short term energy storage, another small reservoir can be built close to the river, however, water from the lower reservoirs would not be exchanged with the river water to maintain the water quality in the SPHS reservoir. |
| **Flood control** | - SPHS plants can be used in combination with conventional flood control mechanisms to improve their efficacy. This combination consists of allowing the reservoirs on the main river to operate with low levels as the long term storage is provided by the SPHS reservoir parallel to the river. So that when the flooding river flows reach the dam in the river, it will be nearly empty and can store large parts of the flood waters. This combination guarantees that the system will store water and energy seasonally and that the dam in the river will have available storage volume to contain the flood. |
| **Transport with waterways** | - The improvement in water management resulting from a SPHS plant would reduce the chances that a waterway runs out of water. |
| **Interbasin Transfer** | - SPHS projects can be combined with an interbasin transfer project to increase the water security of a region or provide balancing between watersheds. |
| **Groundwater recharge** | - The increased regulation of the river flow contributes to a continual supply of water. This could improve groundwater recharge as irrigation channels utilization would increase. |
| **Water security** | - Increase the water storage capacity in regions where conventional storage reservoirs are not appropriate. |

**Supplementary Table 2**

**Assumptions, uncertainties, limitations and possible improvement of the model.**

| Title | Definition |
| --- | --- |
| Assumptions | |
| Dams in cascade | The model assumes that the energy storage potential is not only limited to the upper and lower reservoirs of the SPHS project. It also includes the energy generated at the dams in cascade downstream the SPHS plant. It is assumed that 60% of the hydropower head downstream of the SPHS plant is developed. |
| Land costs | The model assumes that the land cost required for the reservoir is equal to 41,000 US$ ha^-1^. One of the most relevant drawbacks for the construction of SPHS projects is the need to create a large reservoir. Setting a high land cost forces the model to look for projects with small flooded areas and, thus, lower social and environmental impacts. A fixed land cost was used because different countries have different policies involving the construction of reservoirs, which proved to be difficult to quantify in the model. A fixed and high land cost assumes that the costs of appropriate social and environmental measures in the construction of the SPHS reservoir are included in the project cost. |
| Latitude corrections | The model corrects the variation of the resolution of the gridded data according to the latitude. This correction is done in steps of 5^o^ of latitude, according to the segmentation of the topographic data. |
| Reduce Flow Seasonality | In order to create the model, a methodology to estimate how much water could be extracted from the river had to be created in a global scale. Given that the paper intends to resolve seasonal energy and water variation needs, the reduction in river seasonality was selected as a measurement of the amount of water that could be extracted from the river. This might not be the preferred option in the management of the water resources of a basin. For example, in Central Asia it is desired that the river flow continues with the least disturbance from hydroelectric dams because the demand for water in the countries downstream happens during the summer for irrigated agriculture. During the winter the demand for water is low. Other reasons why a reduced flow seasonality is not appropriate are due to environmental restrictions, river mouth and coastal sedimentation and other aspects. |
| River water extraction | There are several models for global scale river network extraction^12^, however, the authors created a methodology to estimate the extraction of water from the river as they could not find in the literature a methodology that could be effectively applied to SPHS plants, that (i) took into account the seasonal and inter-annual variation in a simple and comprehensive manner, (ii) could be applied on a global scale, (iii) guarantee that there would be enough water available to be pumped to the upper reservoir, (iv) to estimate a conservative upper reservoir capacity, which could fill up with the limited pumping capacity, (v) increase the viability of the construction of a SPHS plant, (vi) reduce the risks that the SPHS plant would not have enough water to operate. Another important aspect that was considered by the authors was the impacts of the SPHS plant in the basin hydrology. We compared three possible scenarios for the extraction of water from the river with high extraction (around 50% of the annual river flow), medium (30%) and low (10%). This design decision depends on policies developed by the stakeholders involved in the water management of a basin. Given that the model methodology was to be implemented on a global scale and that it would be complex and controversial to vary this assumption from basin to basin, we decided to be conservative in the amount of water that could be extracted from the river and selected the low extraction scenario. The final extraction is not 10%, but 11% due to the methodology to include seasonal and inter-annual variation indexes in the analysis. For example, if the seasonal and inter-annual variation indexes are equal to 1, then the Equation 3 turns out to be Q_A_ = Q x 0.1 x 1.1 = 0.11, hence the limit of 11% for water extraction from the river. |
| Economic assumptions | The interest rate assumed for the investment for the construction of the project is 4.5%, the plant is estimated to operate for 40 years, the discount factor is 18.2, cost assumptions such as capital, O&M, procurement, uncertainties, construction management, pre-engineering, equipment costs and other costs vary for each component of the project and are presented in^13^.The conversion rate for the Norwegian Krone to US$ was taken from Google on the 08/14/2017 and is assumed to be 0.13. The interest rate used to bring cost from 2010 to 2019 is assumed to be 1.5% per year. |
| Comparison between storage capacity and cost | The comparison between the storage capacity and its cost is divided into the three sections below: (i) if the storage capacity is smaller than the water available to be stored, the costs for water and energy storage consists of the costs for the construction of the project in US$ divided by the amount of water or energy that the project stored throughout the operation of the plant in km^3^ and MWh, respectively. The costs and services that happen throughout the operation of the plant is brought to today’s values with a discount factor of 18.2. (ii) if the storage capacity of the reservoir is smaller than the yearly amount of water available to store times two. Any additional storage capacity that exceeds Q_A_ will only reduce the water and energy storage costs by half when compared to Q_A_ (Equation 4). This is because the section of the reservoir destined to store energy seasonally has a higher chance to fill up than the section destined to store energy inter-annually. (iii) if the storage capacity is bigger than twice the available yearly water availability, W_S_ is fixed and equal to 1.5Q_A_. That means that any increase in storage capacity that surpasses twice the value of the yearly water availability will not contribute to lower the cost of water storage. Note that the energy lost with the storage process is not included in the costs of the project and should be included when designing the plant. The electricity cost for pumping was not included because the costs of electricity and regulations for the operation of storage plants vary considerably from country to country. The energy efficiency assumed for optimizing the costs of the tunnels is 70% (a conservative value). |
| Water and energy storage cost thresholds | The water and energy storage cost thresholds were set with the intention of reducing the total number of projects presented in Fig. 3 to around a thousand, for a cleaner presentation of the results and to focus on possibly viable projects. The threshold cost of water storage of 0.2 US$ m^-3^ is based on the same order of magnitude of seawater desalination of 1 US$ m^-3^. However, a SPHS plant still requires electricity to store the water, which is not included in the 0.2 US$ m^-3^ cost. The threshold cost of energy storage of 50 US$ MWh^-1^ is used based on the same order of magnitude of gas based thermoelectric generation, which varies from 10-50 US$ MWh^-1^. However, note that a gas power plant generates electricity, while a SPHS plant only stores energy. |
| Uncertainties | |
| Segmented topography | The topography utilized in the study was divided into 5 degrees sections. Dams, reservoir and tunnels that reach the borders of the topography file are discarded. |
| Hydrology data | There might be some uncertainties originating from the hydrological data. As the GRIN and PCR-GLOBWB data have different resolution, a methodology was created to increase the resolution of the PCR-GLOBWB data. This methodology consists of giving a single hydrological flow for each river Strahler stream order higher than 7 in each 5 degrees section. This is performed by finding the highest river Strahler stream order of each PCR-GLOBWB 6 min resolution, then taking an average of the hydrological flows for each river Strahler stream order number. A drawback of this methodology is that the river flow for each Strahler stream order in a 5-degree section will be constant. However, errors involving the topographic difference between the data are minimized. In order to improve the results using this methodology, it could have been applied to smaller sections of 1 degree or less. Only rivers with a Strahler stream order above 7 were considered, as they have enough flow to justify the construction of a SPHS plant. There are not many reservoirs that can be filled completely with the water available for extraction. In other words, there is usually more water available than storage capacity. Thus, the water availability is not the main limitation for SPHS potential, but the topography. |
| Cost estimation | The cost estimates in this paper intend to give a rough estimate for project costs of the main components such as dam, tunnel, penstocks, generators, turbine, powerhouse excavations and land requirement. Some cost aspects are not included in these estimates, for example, builder costs (which mainly affect dam costs), access tunnels, transport facilities, substations, transmission lines, shafts, surge chambers, gates, valves, social-environmental and cultural costs, lifetime costs, etc. Some of these costs vary considerably with the country and local characteristics. The cost for tunnel excavation assumes an average value and does not include the type of rock or earth being excavated. The number and diameter of the tunnels are optimized with the minimization of the capital and operation costs, assuming a capacity factor of 70% for the utilization of the tunnel and an electricity price of 65 US$ MWh^-1^. |
| Geological formation | This work does not include the geological composition and structure of the topography. An appropriate geological formation is crucial for the construction of SPHS reservoirs. This is because SPHS reservoir suffers from great pressure due to the high column of water that could be as high as 250 meters in the model. Another issue is that this stress varies throughout the year with fluctuation in level of the reservoir, which could result in fatigues in the geological formation. Thus, the locations considered for the construction of SPHS reservoirs should not have geological fractures and should have a composition that can withstand these pressures and fatigues. |
| Resolution | The resolution of the topography was reduced from 3 sec to 15 sec to increase the computational speed of the model. With a resolution of 15 sec, the model took one month to finish calculations. With a resolution of 3 sec, authors estimate that it would take more than two years to estimate the world potential for SPHS. A resolution of 15 sec is equivalent to 450 meters at the equator, this low resolution impacts mainly the estimation of the SPHS dams. 450 meters is similar to the length of a medium sized dam. This low resolution fails to consider the complete profile of medium sized dams and frequently reduces the estimate of the height and length of the dam required to create the reservoir. This underestimates the costs of the dams for SPHS projects, particularly for dams with lengths shorter than 450 meters. This underestimation, however, reduces with the increase in dam lengths. Aside from dam size, costs are sensitive to the tunnel length. But overall, tunnel lengths are much longer than the topographical resolution so the error is small compared to total tunnel cost. Further explanation on the impact of the topographical resolution on PHS projects can be seen in^14,15^. The hydrological resolution of 6 mins is acceptable because the flow variations per pixel within a river are a small fraction of the total river flow. |
| Limitations | |
| Tunnel design | The tunnel design in the model is limited to the connection of the river with the middle of the dam. Even though this is usually the best design for the tunnels, this might not be always the design of the tunnel. The tunnel could connect to the river, leaving from any point below the minimum reservoir level. |
| Unique dam | The SPHS projects in this model are limited to a single dam. This design covers the majority of the potential. However, there are locations where two or more dams would be required to build a reservoir or to increase its storage capacity. |
| Minimum reservoir level | The minimum reservoir level in this model assumes that all the reservoir volume would be used. This practice is not appropriate and a minimum reservoir level should be left in the reservoir due to environmental restrictions. These restrictions changes with the country. |
| Single SPHS project | The model only creates one SPHS project in parallel to the main river. A cascade of two or more SPHS projects could be created to increase the potential energy of the water and increase the energy and water storage potential. |
| Open-Loop SPHS | Only open-loop SPHS plants are considered in this model, which involve the extraction of water from a river. The river can have a medium or small reservoir to provide a level of regulation in the river flow to guarantee that the SPHS will have enough water to pump to the upper reservoir. Alternatively, if the impacts of building a reservoir in the main river are high, which impacts the viability of the project, a lower reservoir for the SPHS system could be built close to the river. This lower reservoir would then minimize the impact of the operation of the SPHS plant in the river flow. |
| Closed-Loop SPHS | Closed-loop SPHS power plants are not considered in this paper. They usually require two large reservoirs and dams, and only store energy. They do not have a substantial impact on the nearby rivers because they are not built on a river and the water inflow from a river only intends to complement the water losses due to evaporation. The inclusion of closed-loop SPHS in this study would considerably increase the world potential of energy storage with SPHS plants. Even though this arrangement would allow projects to be built far from major rivers, the need for building two large dams and reservoirs close to each other reduces the number of locations with appropriate topography which would make such projects viable. Additionally, there would be no hydropower plants in cascade that would contribute to the increase in energy storage without additional costs. |
| SPHS in series | There is also the possibility of building two SPHS reservoirs in series, where one of the reservoirs is connected to the main river (intermediate reservoir) and the other reservoir is connected to the intermediate SPHS reservoir (upper reservoir). This arrangement is interesting because it increases the total generation head of the plant. Another limitation that this arrangement resolves is the fact that SPHS projects are usually limited to a generation head of 1200 meters. Having two SPHS in series could increase the overall generation head of the plant to 2400 meters. One example of such arrangement can be seen in the Limberg II SPHS plant in Austria. This possibility would considerably increase the potential for water and energy storage presented in this paper, particularly the energy storage potential, due to the increase in overall generation head. |
| Hydropower potential | Even though the tributary river flow is able to fill up the reservoir without the need for pumping water from the main river in three over more than a thousand SPHS plants, as shown in Fig. 3e, the model does not add the additional hydropower generation from the tributary river where the upper reservoir of the SPHS is built. This is because it is complicated to compare hydropower and energy storage. Hydropower generates electricity and SPHS stores energy. Including both alternatives in the model would confuse the analysis. |
| Energy and water complementarity | The needs for energy and water storage with SPHS plants should be complementary. This is because during the dry season there will be low volumes of water available to be used for energy storage. This complementarity is usually the case in high latitude countries, where during the summer river flow is higher due to ice melting and energy demand is lower compared to the winter. Inter-tropical regions with abundant hydropower generation also have complementarity, where during the wet season there is high water availability and hydropower generation. However, there are regions and countries where the need for energy and water storage is not complementary, for example, in the inter-tropical regions without hydropower generation, where the summer and wet season is the period with highest electricity demand due to air conditioning. Other cases of lack of complementarity can happen in locations where the dry season has higher solar power generation, or wet season with low solar power generation, or high electricity demand and flooding risk, etc. In cases where energy and water storage need are not complementary, SPHS should not be considered as an energy and water storage alternative. Exceptions can be made to SPHS projects that have heads higher than 800 meters, in which low volumes of water can store large volumes of energy. |
| Energy and water needs | The needs for energy and water storage are not included in this paper and are an important aspect when planning a SPHS plant. |
| Restriction zones | The model assumes only technical aspects of SPHS projects. Restrictions such as biodiversity, population resettlement, social opinion, historical buildings and locations, environmentally protected areas, conflict zones were not included in the analysis. These restrictions would have a considerable impact on the estimated capacity and costs for water and energy storage. Further studies should be implemented and include considerations for the restriction mentioned above and other restrictions. |
| Lakes | Existing lakes are not included as possible lower reservoir possibilities. The advantage of using a lake as the lower reservoir is because there are much smaller restrictions from pumping water from a lake, when compared to a river. The inclusion of lakes to the model would considerably increase the world potential of SPHS. |
| Model parameters limitations | The model was designed to have the least possible restrictions. However, the model was taking too long to converge. Thus, we restricted the value of some parameters to reduce computational time. The run presented in this paper took one month to converge with a desktop with a processor Intel Core i7-6700, 3.4 GHz x 2. The main restrictions are the (i) length of the dams of 7.2 km, (ii) size of the reservoirs of 1.620 km^2^, (iii) dam heights of 50 to 250 m, and (iv) tunnel length of 3 to 30 km. The maximum dam length and reservoir area were set high with the expectation that they [and values over and above these maxima] would not result in viable projects, that are cheaper than 0.2, US$ m^-3^ for water storage and 50 US$ MWh^-1^ for energy storage. It turns out that there are viable projects with dam lengths higher than 7,2 km, but no viable projects with flooded areas higher than 1,620 km^2^. If we would run the model again, we would increase the dam length to 14.4 km. |
| Other information | |
| Water and energy projects | There are 5.1 million water and energy projects considered. From these projects, their water and energy storage costs are estimated. It turns out that for each 1 arc degree resolution there are 1,457 projects with water storage costs lower than 0.2 US$ m^-3^ and 1,092 energy projects with energy storage cost lower than 50 US$ MWh^-1^. Some water and energy projects presented in Fig. 3 consist of the same project. However, most of them are different because cheap energy storage projects usually have high heads. The higher the head, the more energy the SPHS plant stores. Water storage projects, on the other hand, are not affected so much by the head variation between the lower and the upper reservoir. |
| Multiple Complementary Services | Each project can be used to provide multiple and complementary services, however, it would be confusing to combine both services in the paper as each location was different water and energy storage needs. Thus, we decided to present the water and energy storage costs individually. If the SPHS plant is used for energy and water storage, the cost of energy and water storage will reduce as both services will contribute to the viability of the project. |
| Installed Capacity | This paper assumes that all projects proposed have 1 GW of installed capacity. The cost of the installed capacity of the SPHS plant in Fig 3d only includes the costs of the tunnels, turbine, excavation and electric equipment, excluding the dam and land cost. This is purposely set to allow the reader to design their own SPHS projects. For example, if the reader wants to propose a project with 2 GW, they will use the cost for an energy storage project in Fig 3c and add the cost in Fig 3d to the project. Alternatively, if the user wants to develop a project with 500 MW, he or she will deduct half the cost presented in Fig 3d to the project. |
| Comparison with other short term energy storage technologies | Currently, PHS is the most economical alternative for short term energy storage, however, with the reduction in the cost of batteries it is possible that batteries will become cheaper than PHS plants. SPHS can provide both long and short term storage services. This combination of services could further increase the viability of SPHS for short term storage, due to the provision of two services with one plant. On the other hand, SPHS plants with short heads or long tunnels, i.e. with high installed capacity costs per GW, might not be able to compete with the cost of short energy storage provided by the batteries. In these cases, the operation of SPHS plants would focus on storing energy only for the long term and in crucial moments of the grid when peak generation is required. |
| Model Validation | We validated different aspects of the model separately. For example, ^14,15^ also used SRTM topographic data to estimate the volume of their reservoirs and validated their reservoir storage capacity with a sample of reservoirs or lakes with known volumes, and showed that the error in estimating the water storage was small. The reference from where the costs analysis was taken from^13^ is validated by the extensive experience of construction of hydropower plants in Norway. The final costs for energy storage with PHS are similar to costs presented in the literature of 400 to 800 € kW^-1^ and 5 to 150 € kWh^-1 16^. Another approach to validate the model is to visually check Supplementary Fig. 3 if the proposed SPHP projects follow the model specifications. |
| Possible improvement | |
| Conservative water storage potential | In very dry locations, availability of water is restricted to a few weeks or months. In these cases, it might be advisable to store up to 50% of the river flow, so that the resource can be appropriately managed. |

**Supplementary Table 3
Description of the data and methods applied in the model.**

| Data and methods description | Available resolution | Utilized  resolution | Comments | Links in  the paper | Reference |
| --- | --- | --- | --- | --- | --- |
| Topographical data (SRTM) | 3 sec  90 x 90 m^*^ | 15 sec  450 x 450 m^*^ | Topographic data for latitudes between 60°N to 56°S.The reduction in resolution assumes the central point of 15 sec of the 3 sec data. | Fig. 1a | ^17^ |
| River Network, Strahler data (GRIN) | 15 sec  450 x 450 m^*^ | 15 sec  450 x 450 m^*^ | This data is derived from the same topographical data above. This is used to give a better estimate of the tunnel length connecting the river and the reservoir. | Fig. 1b | ^12^ |
| Hydrological data (PCR-GLOBWB) | 6 mins  10.8 x 10.8 km^*^ | 6 mins  10.8 x 10.8 km^*^ | This data combines the estimated water availability and use over the period 1960–2010 and includes human activity. More details on the data can be seen in reference^18^. The annual discharge, seasonal and inter-annual variation are derived from this data. As the GRIN and PCR-GLOBWB data have different resolution, a methodology was created to increase the resolution of the PCR-GLOBWB data. This methodology consists of giving a single hydrological flow for each river Strahler stream order higher than 7 in each 5 degrees section. This is performed by finding the highest river Strahler stream order of each PCR-GLOBWB 6 min resolution, then taking an average of the hydrological flows for each river Strahler stream order number. A drawback of this methodology is that the river flow for each Strahler stream order in a 5-degree section will be constant. However, errors involving the topographic difference between the data are minimized. In order to improve the results using this methodology, it could have been applied to smaller sections of 1-degree or less. Assuming that there are uncertainties associated with the PCR-GLOBWB global hydrological model, the error of this methodology is small. Only rivers with a Strahler stream order above 7 were considered, as they have enough flow to justify the construction of a SPHS plant. | Fig. 1g | ^18^ |
| Pumped Storage Costs | - | - | This reference gives detailed data on pumped-storage costs, such as dam, tunnels, excavation, electrical equipment and turbine costs. The model assumes most cost estimates proposed by the reference^13^. It also assumes only one type of construction design for each of the components of the SPHS plant. This is because, it would be complex to create a model that compares different designs for each component to find the most optimum one. This gave a good preliminary estimate of the final costs. For the construction of the dam, the model assumes a rockfill dam with central moraine sealing, as described in Fig. B.1.1^13^. For the construction of the tunnels it assumes drill and blast, as described in Fig. B.1.4^13^. The penstock costs include the costs of digging the tunnel (Fig. B.9.2^13^) and the cost of the embedded steel pipes (Fig. M.6.C^13^). The excavation varies with the generation head and the installed capacity, as described in Fig. B.10.1^13^. The turbine assumed is Francis, as described in Fig. M.1.b^13^ and Fig. M.4.A^13^. The selection of the turbine also depends on the generator, as described in Fig.E.8.2.a^13^. For the optimization of the turbine/generator system, the costs of different rotation speeds, as described in Fig. E.1.1a^13^ and Fig. E.8.1.b^13^, are compared to the average generation head and flowrates under analysis and the cheapest option is selected. Note that one turbine/generator system is proposed per tunnel. | Estimate Project Cost Stage | ^13^ |
| Tunnelling Design | - | - | The methodology used to optimize the construction of the tunnels was taken from^19^. This methodology consists of comparing the capital costs of construction of the tunnels, such as the diameter and number of tunnels, and the costs of operating the plants. The cost of operating the plants depends considerably on the energy losses due to friction in the tunnels. The bigger the diameter and number of tunnels the more efficient is the plant. | Estimate Project Cost Stage | ^19^ |

* Distance at the equator, which is corrected with changes in latitude.

**Supplementary Table 4
Seasonal-pumped storage world potential model stages description.**

| Model stages | Description | Links in the paper |
| --- | --- | --- |
| Select Point Under Analysis (PUA) | This section consists of combining topographic and the river Strahler data and going through each land grid square around the world looking for SPHS projects considering the limitations presented in this paper. | Fig. 1a |
| River Screening | This stage looks for a river with a reasonable amount of water to store. It makes sure that the SPHS upper reservoir is not in the same river as the lower reservoir, i.e. it is a parallel river. If finds rivers with Strahler stream order higher than 7 at a distance from 3 to 30 km distance from the Point Under Analysis (PUA) and the model continues. If there are rivers with different river Strahler stream order of 7 to 12 at less than 30 km from the upper reservoir the model will create a different SPHS project for each river. | Fig. 1b |
| Dam Screening | This stage creates four different dams in the given orientations: W to E, N to S, NE to SW, NW to SE. The dam height varies from 50 to 250 m, at 50 m intervals. Each grid square can have projects with five different dam heights. | Fig. 1d |
| Dam Lowest Point | In order to reduce the number of interactions, this stage checks if the pixel under analysis is the lowest point of the proposed dams. If it is the lowest point of the proposed dams, the model continues developing the SPHS project. This grid cell usually coincides with a tributary river. | Fig. 1e |
| Reservoir Side and Flooding | This stage checks which side of the dam should be flooded to build the reservoir. If the reservoir floods an area larger than 1,620 km^2^, then the model floods the other side of the dam. If both flooded areas are larger than 1,620 km^2^, then the project is discarded. | Fig. 1f |
| Reservoir Storage Capacity | Once the storage reservoir is flooded, the level of the reservoir varies to find the flooded area vs level and storage volume vs level curves. This is done by subtracting the volume of land and water with the reservoir at a given level by the volume of land and water within the reservoir at its minimum level. In the model the minimum level of the reservoir is assumed to be zero. | - |
| Reservoir, Hydrology Comparison | The hydrology is included in the analysis to limit the water and energy storage capacity of the SPHS projects according to the availability of water in the main river. The maximum water storage capacity is limited to 11% of the river flow. If the storage capacity is much higher than the amount of water available, the estimated cost of storage tends to zero, as the reservoir will never fill up. In other words, this section does not remove the project that does not have enough water to fill up the reservoir. It calculates the cost of energy and water storage with a large reservoir, even if the water available in not enough to fill the reservoir. For example, if the reservoir is two times larger than the water available, then the cost of energy storage will be higher than if there was enough water to fill the reservoir. Thus, the reservoir becomes too expensive and is not selected. The same reservoir with a smaller dam is selected instead, as the cost of the dam and flooded area are smaller. In other words, the project is not cancelled it is just not selected. | Equation 1  Equation 2 Equation 3 |
| Estimate Project Cost | This section calculates the project costs, which are divided in dam, tunnel, powerhouse excavation, pump-turbine, electro-technical equipment and land costs. | Supplementary Tables 4 and 7 |
| Estimate Storage Cost | The project costs are compared with the hydrology of the river to find the water and energy storage costs. | Equation 4  Equation 5 |

**Supplementary Table 5
SPHS project parameters analysed and average value of selected projects.**

|  | Minimum possible | Intermediate steps | Maximum possible | Minimum  observed | Maximum  observed | Average for water storage projects | Average for energy storage projects |
| --- | --- | --- | --- | --- | --- | --- | --- |
| Dam height (m) | 50 | 50 | 250 | 50 | 250 | 140.7 | 151.7 |
| Dam length (km) | 0.32 | 0.32 | 7.20 | 0.32 | 7.20 | 1.70 | 1.65 |
| Tunnel length (km) | 2 | 3 | 30 | 3 | 30 | 22.2 | 22.7 |
| Land requirement (km^2^) | 0.2 | 0.2 | 1,625 | 3.3 | 1,366.4 | 34.2 | 36.4 |
| River Strahler stream order | 7 | 1 | 12 | 7 | 11 | 7.48 | 7.44 |
| Minimum head (m) | 25 | - | 1,200 | 134.5 |  | 478.3 | 602.0 |
| Level variation (m) | - | - | - | 50 | 250 | 140.6 | 151.7 |
| Storage volume (km^3^) | - | - | - | 0.20 | 56.89 | 1.89 | 1.65 |
| Energy storage (TWh) | - | - | - | 0.93 | 495.52 | 7.21 | 8.33 |
| Land use / water storage (km^2^ km^-3^) | - | - | - | 5.6 | 68.2 | 19.7 | 21.2 |
| Land use / energy storage (km^2^ TWh^-1^) | - | - | - | 0.40 | 209.4 | 8.31 | 6.31 |
| River discharge (m^3^ s^-1^) | - | - | - | 47.8 | 50,604.0 | 1,344.9 | 1,149.8 |
| Seasonality variation index S_V_ from Eq. 1 | - | - | - | 0.04 | 2.22 | 0.76 | 0.80 |
| Inter annual variation index I_v_ from Eq. 2 | - | - | - | 0.03 | 5.80 | 0.57 | 0.56 |
| Pumping flow (m^3^ s^-1^) | - | - | - | 94.5 | 842.1 | 267.5 | 213.2 |
| Dam cost (B US$) | - | - | - | 0.044 | 6.749 | 0.653 | 0.655 |
| Tunnel cost (B US$) | - | - | - | 0.100 | 39.606 | 0.881 | 0.696 |
| Excavation cost (B US$) | - | - | - | 0.051 | 0.073 | 0.059 | 0.059 |
| Pump-turbine cost (B US$) | - | - | - | 0.081 | 0.125 | 0.090 | 0.088 |
| Electrotechnical equipment cost (B US$) | - | - | - | 0.168 | 0.201 | 0.171 | 0.170 |
| Land cost (B US$) | - | - | - | 0.0151 | 6.181 | 0.155 | 0.165 |
| Water storage cost (US$ m^-3^) | - | - | - | 0.007 | 0.2 | 0.104 | 0.133 |
| Energy storage cost without cascade (US$ MWh^-1^) | - | - | - | 4.606 | 50.0 | 35.412 | 30.139 |
| Energy storage cost with cascade (US$ MWh^-1^) | - | - | - | 1.832 | 50.0 | 31.467 | 24.841 |
| Energy storage costs (US$ kW^1^) | - | - | - | 372 | 600 | 439 | 459 |

**Supplementary Table 6**

**Selected project parameters change with dam height in Tibet, China.**

| Dam height (m) | Dam length (km) | Land req. (km^2^) | Water storage  (US$ km^-3^) | Water Storage volume (km^3^) | Energy storage without cascade  (US$ MWh^-1^) | Energy Storage without cascade (TWh) | Energy storage with cascade  (US$ MWh^-1^) | Energy Storage with cascade (TWh) |
| --- | --- | --- | --- | --- | --- | --- | --- | --- |
| 50 | 0.40 | 13.0 | 0.069 | 0.60 | 34.8 | 1.19 | 11.66 | 3.56 |
| 100 | 1.21 | 15.8 | 0.037 | 1.31 | 18.0 | 2.68 | 6.16 | 7.86 |
| 150 | 1.21 | 16.7 | 0.034 | 2.12 | 16.3 | 4.46 | 5.66 | 12.80 |
| 200 | 1.21 | 19.5 | 0.036 | 3.02 | 16.8 | 6.53 | 5.96 | 18.42 |
| 250 | 1.21 | 22.1 | 0.046 | 4.03 | 20.8 | 8.97 | 7.52 | 24.85 |

**Supplementary Table 7**

**Selected project parameters that do not change with dam height.**

| Coordinates (lat/lon) | Min.  head (m) | Tunnel length (km) | River Strahler stream order | River discharge (m^3^ s^-1^) | Pumping flow  (m^3^ s^-1^) | River seasonality index S_V_ from Eq. 1 | River Inter Annual Var. index I_v_ from Eq. 2 | Short-term Energy storage  (US$ kW^-1^) |
| --- | --- | --- | --- | --- | --- | --- | --- | --- |
| N 28^o^43’43”  E 97^o^02’44” | 782 | 18 | 7 | 526 | 145 | 0.88 | 0.24 | 0.65 |

**Supplementary Fig. 1**

**Water used for storage in SPHS projects.** The blue dots represent the proportion of water that each of the SPHS projects extracts from the river. As can be seen the withdrawal is constrained to 11% of the total flow of the river, although for many larger rivers the withdrawals can be significantly less.


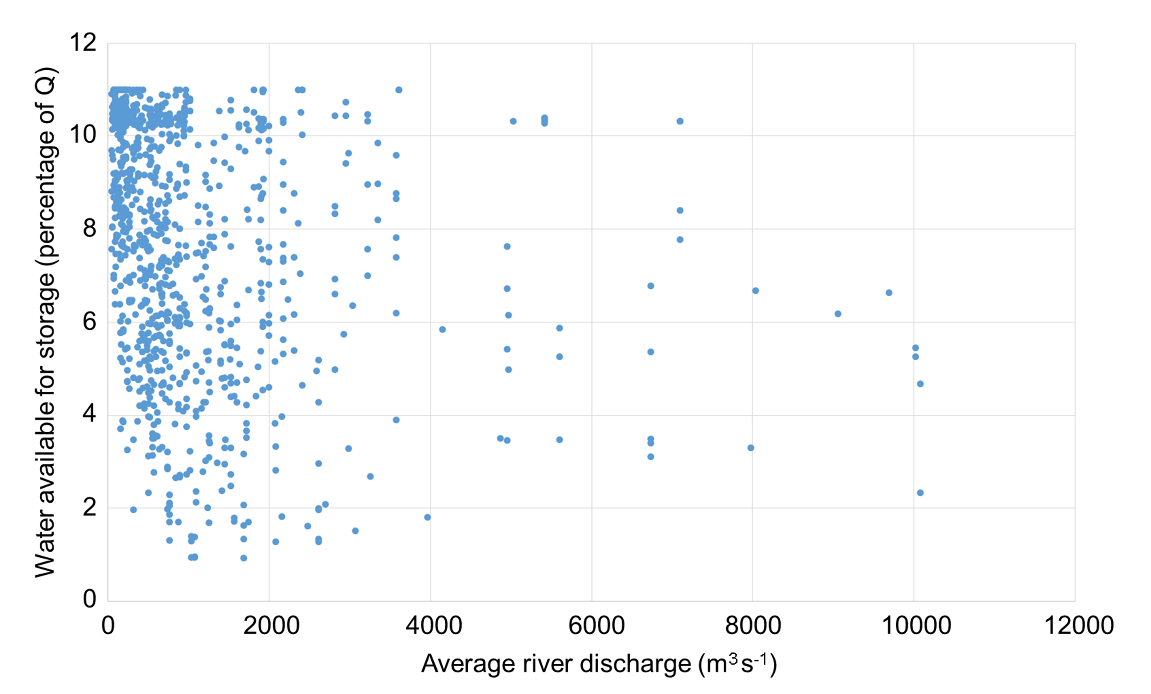


**Supplementary Fig. 2**

**Seasonal and inter annual variation. a,** accounts for all SPHS projects in each basin and calculates the average seasonal variation of the rivers connected to the SPHS reservoir. **b,** Average inter-annual variation.


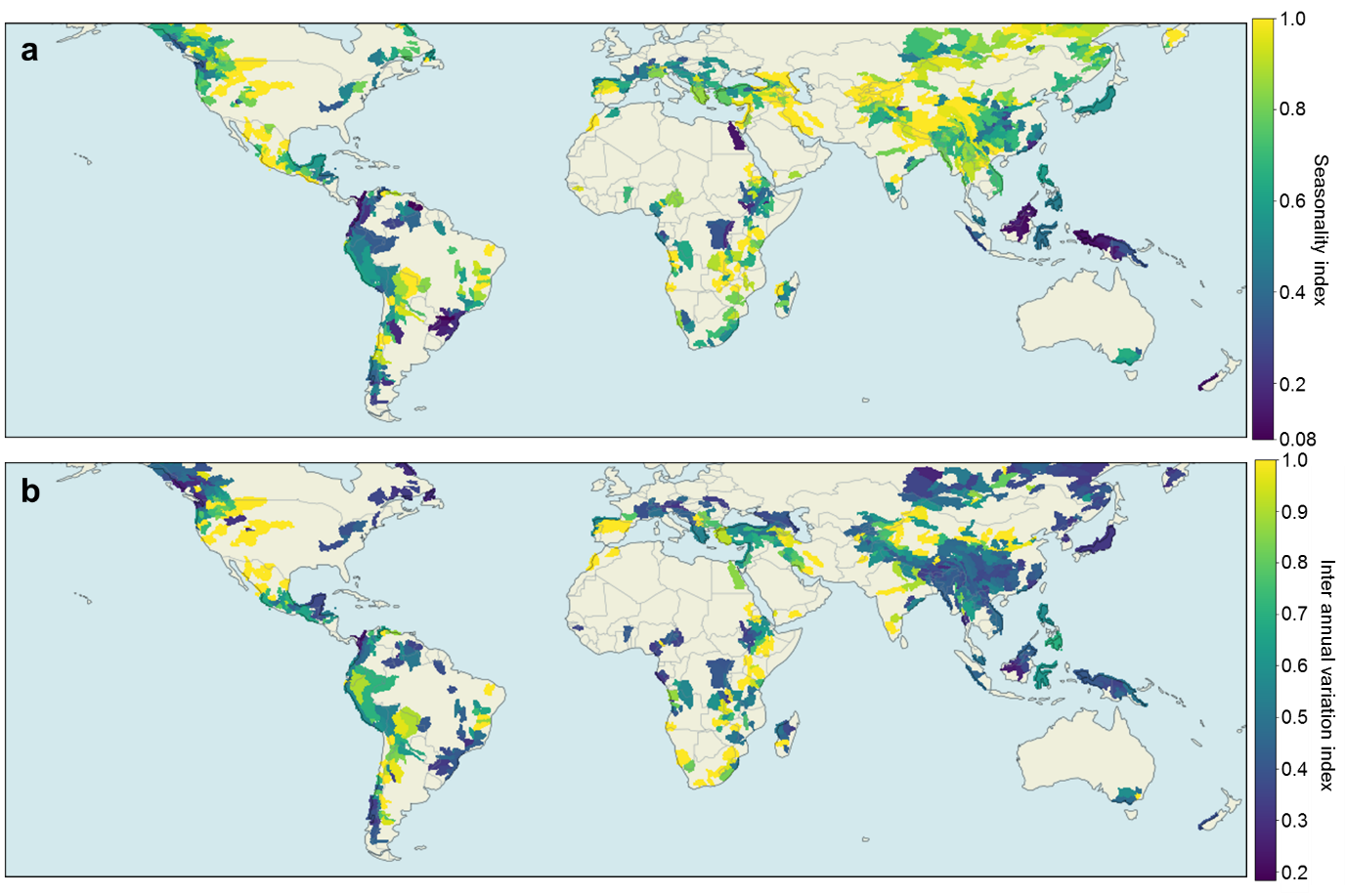


**Supplementary Fig. 3**

**Proposed SPHS pipelines.** The white lines correspond to the pipelines proposed in the SPHS world potential model in different locations. The proposed projects are presented above a topographic background of the locations. Note that from these projects only the cheapest in 1 degree resolution are considered in the paper.


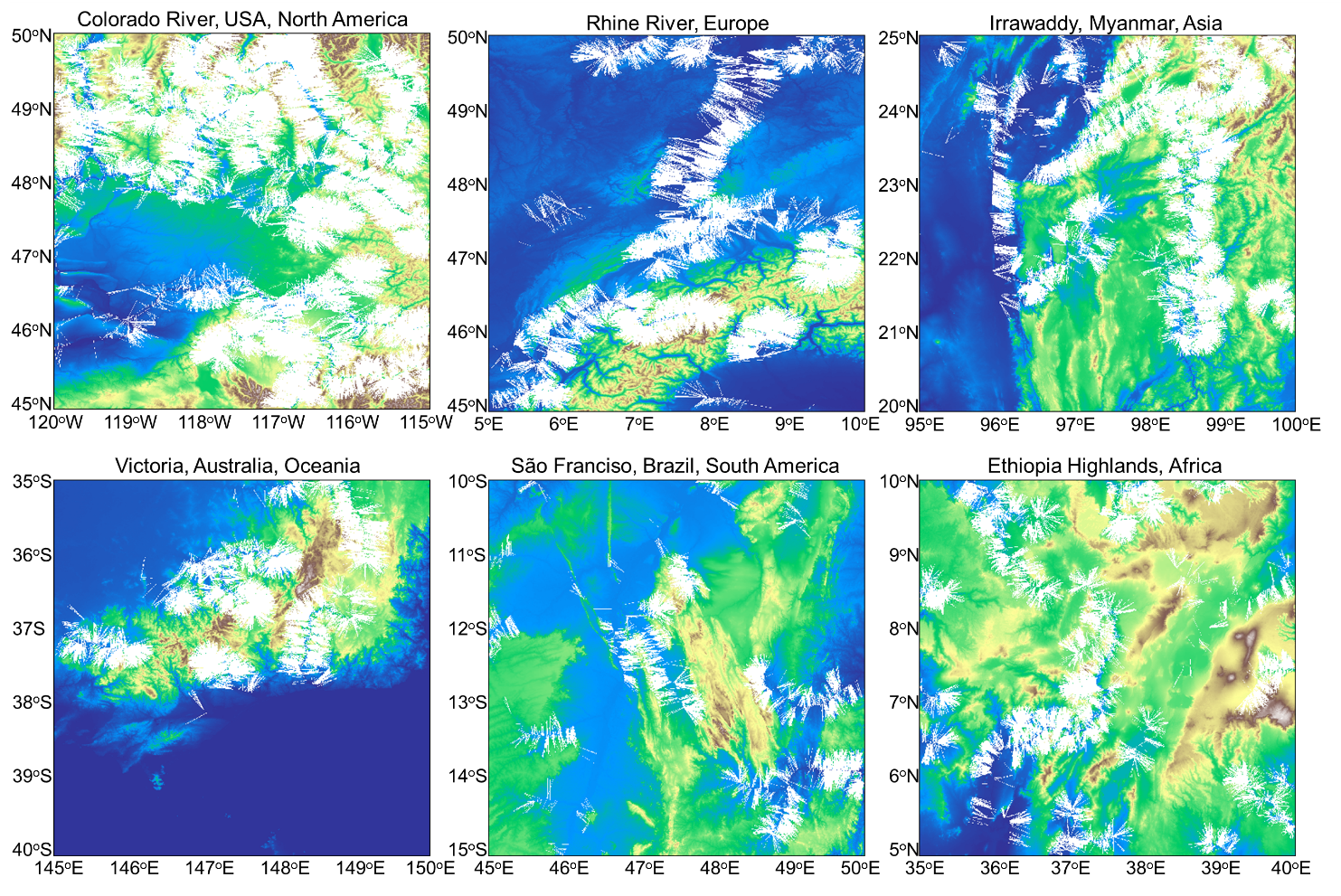


**Supplementary References**

1. Hunt, J. D., Freitas, M. A. V. D. & Pereira Junior, A. O. A review of seasonal pumped-storage combined with dams in cascade in Brazil. Renew. Sustain. Energy Rev. 70, (2017).

2. Hunt, J. D., Freitas, M. A. V. & Pereira Junior, A. O. Enhanced-Pumped-Storage: Combining pumped-storage in a yearly storage cycle with dams in cascade in Brazil. Energy 78, (2014).

3. Olauson, J. et al. Net load variability in Nordic countries with a highly or fully renewable power system. Nat. Energy 1, (2016).

4. International Energy Agency & Shin-Ichi Inage. Prospects for Large-Scale Energy Storage in Decarbonised Power Grids. (2009).

5. Converse, A. Seasonal Energy Storage in a Renewable Energy System. Proc. IEEE 100, 401–409 (2011).

6. Pehl, M. et al. Understanding future emissions from low-carbon power systems by integration of life-cycle assessment and integrated energy modelling. Nat. Energy 2, 939–945 (2017).

7. International Energy Agency. Energy Technology Perspectives: Scenarious & Strategies to 2050. (2008).

8. Conway, D., Dalin, C., Landman, W. A. & Osborn, T. J. Hydropower plans in eastern and southern Africa increase risk of concurrent climate-related electricity supply disruption. Nat. Energy 2, 946–953 (2017).

9. Bueno, C. & Carta, J. A. Wind powered pumped hydro storage systems, a means of increasing the penetration of renewable energy in the Canary Islands. Renew. Sustain. Energy Rev. 10, 312–340 (2006).

10. Portero, U., Velázquez, S. & Carta, J. A. Sizing of a wind-hydro system using a reversible hydraulic facility with seawater. A case study in the Canary Islands. Energy Convers. Manag. 106, 1251–1263 (2015).

11. Winemiller, K. O. et al. Balancing hydropower and biodiversity in the Amazon, Congo, and Mekong. Science (80-. ). 351, 128–129 (2016).

12. Schneider, A., Jost, A., Coulon, C., Silvestre, M., Théry, A. Ducharne, A. Global scale river network extraction based on high-resolution topography and constrained by lithology, climate, slope, and observed drainage density. Geophys. Res. Lett. 44, 2773–2781 (2017).

13. Cost base for hydropower plants (With a generating capacity of more than 10,000 kW). Published by: Norwegian Water Resources and Energy Directorate. Editor: Jan Slapgård. Authors: SWECO Norge AS.

14. Gimeno-Gutiérrez, M. & Lacal-Arántegui, R. Assessment of the European potential for pumped hydropower energy storage based on two existing reservoirs. Renew. Energy 75, 856–868 (2015).

15. Rogeau, A., Girard, R. & Kariniotakis, G. A generic GIS-based method for small Pumped Hydro Energy Storage (PHES) potential evaluation at large scale. Appl. Energy 197, 241–253 (2017).

16. Zakeri, B. & Syri, S. Electrical energy storage systems: A comparative life cycle cost analysis. Renew. Sustain. Energy Rev. 42, 569–596 (2015).

17. Jarvis A., H.I. Reuter, A. Nelson, E. G. Hole-filled seamless SRTM data V4. International Centre for Tropical Agriculture (CIAT) (2008).

18. Wada, Y., Graaf, I. & van Beek, L. High-resolution modeling of human and climate impacts on global water resources. J. Adv. Model. Earth Syst. 8, 735–763 (2016).

19. Rognlien, L. Pumped Storage Development in Øvre. Otra, Norway. MSc Thesis. Norwegian University of Science and Technology (2012).
